# MFAP5 promotes basal-like breast cancer progression by activating the EMT program

**DOI:** 10.1186/s13578-019-0284-0

**Published:** 2019-03-07

**Authors:** Yanmei Wu, Ping Wu, Quan Zhang, Wenjin Chen, Xishui Liu, Weiqiang Zheng

**Affiliations:** 1 Department of Breast Surgery, Changhai Hospital, Naval Medical University, 800 Xiangyin Road, Shanghai, 200433 China; 2 Department of Pathology, Maternal and Child Health Care Hospital, Huaian, 223002 Jiangsu China; 3 Basic Medical College, Naval Medical University, Shanghai, 200433 China; 4 Department of Pathology, Changhai Hospital, Naval Medical University, Shanghai, 200433 China

**Keywords:** MFAP5, Basal-like breast cancer, EMT, TGF-β, Notch

## Abstract

**Purpose:**

Human basal-like breast cancer (BLBC) is an aggressive malignancy with poor prognosis. Since most current treatments are ineffective, there is an urgent need to identify therapeutic targets for BLBC. Microfibrillar-associated protein 5 (MFAP5) plays an important role in the integration of elastic microfibers and the regulation of endothelial cell behaviors. We previously demonstrated that MFAP5 was significantly overexpressed in BLBC tissues and associated with poor metastasis-free survival of patients with BLBC. However, the detailed role of MFAP5 in BLBC is unclear. Thereby, the current study aimed to investigate the underlying function of MFAP5 in BLBC.

**Method:**

Functional analyses were conducted for the role of MFAP5 in BLBC in vitro and in vivo.

**Results:**

Overexpression of MFAP5 resulted in a significant increase in the proliferation, migration, invasion and epithelial–mesenchymal transition (EMT) markers in BLBC in vitro and in vivo. In addition, other metastasis animal models by tail intravenous injection of BT20 cells further confirmed that MFAP5 overexpression promoted BLBC proliferation and BT20 cells metastasis. We found that the TGF-β or Notch inhibitor significantly reversed the tumorigenicity and metastasis of MFAP5-induced BLBC cells.

**Conclusion:**

Our findings suggest that MFAP5 may promote EMT in BLBC metastasis via the TGF-β/Notch pathway.

**Electronic supplementary material:**

The online version of this article (10.1186/s13578-019-0284-0) contains supplementary material, which is available to authorized users.

## Introduction

Breast cancer is the second leading cause of cancer for women mortality worldwide [1]. According to gene expression profiling, it can be classified into four major molecular subtypes: luminal A, luminal B, human epidermal growth factor receptor 2 (HER2) and human basal-like breast cancer (BLBC) [1]. BLBC has low expression of the estrogen receptor (ER), progesterone receptor (PR) and HER2 gene, while the expression of basal cytokeratins (CK5/6, CK14, and CK17), epidermal growth factor receptor (EGFR), c-kit and p53 are transcriptionally upregulated [1, 2]. People suffered from BLBC present with aggressive clinical behaviors, such as high histologic grade, distant metastasis to the lung and brain within 3–5 years, a poor prognosis and short disease-free and overall survival [3, 4]. Currently there is still no targeted treatment for BLBC and the only choice of chemotherapy is not effective as well [5, 6]. Therefore, it is very urgent for us to investigate the underlying molecular mechanisms of BLBC metastatic process and find a new therapeutic target.

Some researches define BLBC for its negative expression of triple-negative phenotype (ER, PR and HER2), but several evidences have demonstrated that BLBC is not synonymous with triple-negative breast cancer [7, 8]. Utilizing additional immunohistochemistry (IHC) markers such as basal cytokeratins and EGFR have proven to be better in defining BLBC than triple-negative phenotype, but the disadvantage is the lacking of accuracy [9, 10]. Thus validation of a diagnostic test and the accurate single marker for identification of BLBC in the clinic remains a bottleneck [6, 11].

Matysiak et al. [12] stated that epithelial–mesenchymal transition (EMT) promoting transcription factors were negative prognostic markers in breast cancer based on a review of current available literatures. During EMT process, a variety of signaling pathways are involved in the activation of EMT such as tumor growth factor-β (TGF-β), nuclear factor-κB (NF-κB), Notch, RTK/Ras, Wnt/β-catenin pathways [13]. Li [14] found that LKB1/AMPK could be used as a target of TGF-β pathway in breast cancer cells to control the development of breast cancer. TGF-β induces cell cycle to arrest in G0/G1 phase, and in the TGF-β signaling pathway STIM1 and store-operated calcium entry (SOCE) play an anti-proliferative role in breast cancer [15]. In addition, more and more evidences have shown that Notch1 overexpression is strongly associated with breast cancer invasion, which is an important factor in maintaining the malignant phenotype of breast cancer stem cells [16]. Notchl signaling also correlates with self-renewal and differentiation of breast cancer stem cells (CSCs) and their malignant behaviors [17].

Microfibrillar-associated protein 5 (MFAP5), previously known as MAGP2, is a multifunctional secretion protein, which plays an important role in the integration of elastic microfibers, regulation of endothelial cell behavior and cell survival [18–21]. Mok et al. [22] had demonstrated that MFAP5 can be used as an independent adverse prognosis predictor via studying of a group of advanced ovarian serous papillary adenocarcinomas. Some genes such as FGF18, FGFR2, ADAM12, NEDD9, MMP13 and CDC2 were also up-regulated in the related signaling pathway, which indicated that MFAP5 is indirectly involved in the regulation of various protein kinase pathways in breast cancer. Other researches also showed that MFAP5 mediates ovarian cancer cell motility and invasion potential through FAK/CREB/TNNC1 signaling pathways, suggesting that it may be a new modality of ovarian cancer treatment [23]. Moreover, some researchers also found that there was a significant correlation between MFAP5 and lymph node metastasis, tumor metastasis and patient’s overall survival in early tongue cancer [24]. This study also pointed out MFAP5 might be a mysterious target to predict the prognosis of cervical cancer and tongue cancer metastasis [24].

In the previous study, we set out to systematically analyze the gene chip containing 48, 804 test genes to identify specific biomarkers for BLBC, and confirmed that the MFAP5 gene was significantly up-regulated in BLBC by RT-PCR verification [25]. We also found that overexpression of MFAP5 was significantly correlated with TNM staging and axillary lymph node metastasis in BLBC, suggesting that the MFAP5 gene plays an important role in the development and metastasis of BLBC. Furthermore, we aimed to explore the relationship between MFAP5 expression and EMT phenotypes and investigate the impact of MFAP5 on the invasion and metastasis of BLBC in vitro and in vivo. Finally, the possible involvement of TGF-β/Notch pathways was also investigated.

## Materials and methods

### Cell culture, antibodies and reagents

Human breast cancer cell lines HS578T and BT20 were purchased from American Type Culture Collection. Cells were routinely maintained in Dulbecco’s modified Eagle’s medium (DMEM, HyClone/Thermo) supplemented with 10% fetal bovine serum (Biowest), 50 IU/ml of penicillin, and 50 µg/ml of streptomycin. Cells were kept at 37 °C in a humidified incubator with 5% CO_2_. Phosphate-buffered saline (PBS) with 0.05% trypsin (Invitrogen) was used for cell harvesting and passage. Antibodies for MAGP2 (MFAP5), Smad2/Smad3 and p-Smad2/Smad3 were purchased from Abcam (Cambridge, UK). Antibodies for E cadherin, N-cadherin, vimentin and Type I Collagen were purchased from Bioss (Shanghai, China). TGF-β inhibitor SB431542 and the Notch inhibitor ly-411575 were purchased from Selleck (USA).

### Plasmid construction and transfection

#### Lentivirus production

MFAP5 related lentivirus plasmid and vehicle were designed and produced by NOVOBIO (Shanghai, China). To overexpress MFAP5, the full-length MFAP5 gene was constructed in the lentiviral vectors PDS087_pL6-TO-V5-GIM to obtained PL6-TO-V5-HOMO-MFAP5 vector. To suppress MFAP5, we designed three pairs of Oligo RNA according to the MFAP5 sequence (MFAP5-109-F: CACCGCGACTCCAGAAACATTCACACGAATGTGAATGTTTCTGGAGTCGC; MFAP5-109-R: AAAAGCGACTCCAGAAACATTCACATTCGTGTGAATGTTTCTGGAGTCGC), (MFAP5-330-F: CACCGCACCAGTTTACGACGTATGTACGAATACATACGTCGTAAACTGGTG; MFAP5-330-R: AAAACACCAGTTTACGACGTATGTATTCGTACATACGTCGTAAACTGGTGC),

(MFAP5-379-F: CACCGCTTGTCTGTAAGGAACACGAACGAATTCGTGTTCCTTACAGACAAG; MFAP5-379-R: AAAACTTGTCTGTAAGGAACACGAATTCGTTCGTGTTCCTTACAGACAAGC). Three shRNAs specific (PL-shRNA-GFP-homo-MFAP5-109, PL-shRNA-GFP-homo-MFAP5-330, PL-shRNA-GFP-homo-MFAP5-379) for the above sequences using PDS019_pL_shRNA_F lentiviral vectors were acquired. The lentiviruses were produced by transiently transfecting individual lentiviral PL6-TO-V5-HOMO-MFAP5 vector, PL-shRNA-GFP-homo-MFAP5-109, PL-shRNA-GFP-homo-MFAP5-330 and PL-shRNA-GFP-homo-MFAP5-379 together with packaging and envelope plasmids into HEK-293T cells using Lipofectamine 2000 (Invitrogen). Viral supernatants were collected and passed through 0.45-µm syringe filters.

#### MFAP5-overexpressing cells

For the transfection, the target human breast cancer cell BT20 were seeded in 6-well plates and allowed to attach overnight. Then, the culture medium was replaced with transfection enhancing solution containing PL6-TO-V5-HOMO-MFAP5 lentivirus (MOI = 20). Stable cell line BT20-LV-MFAP5 with MFAP5 overexpression was selected with 1–2 μg/ml BSD. The overexpression of MFAP5 was confirmed by quantitative reverse transcriptase PCR (qRT-PCR), immunofluorescence and Western blotting.

#### MFAP5-knockdown cells

The target human breast cancer cell HS578T was infected with 40 MOI lentivirus PL-shRNA-GFP-homo-MFAP5-109, PL-shRNA-GFP-homo-MFAP5-330 or PL-shRNA-GFP-homo-MFAP5-379, respectively. The control group was negative control virus infection group and blank cell culture group. Cells were collected after 48 h to extract RNA. The optimal interference lentivirus PL-shRNA-GFP-homo-MFAP5-330 was screened by RT-PCR detection. MFAP5-knockdown stable cell lines HS578T-MFAP5-shRNA were selected with 1–2 μg/ml BSD. The knockdown efficiency of MFAP5 was confirmed by qPCR, immunofluorescence and Western blotting.

### RNA extraction and quantitative real-time PCR analysis

Total RNA was isolated using an RNeasy Mini kit (Invitrogen) according to the manufacturer’s instructions. Reverse transcription was performed with PrimeScript TM Master Mix (Invitrogen) according to its product manual. Then, the qPCR was performed with SYBR Premix EX TaqTM II (Invitrogen) according to the manual on the CFX96TM Real-Time detection System (BioRad). β-actin was used as the reference, and the data were analyzed with a normalized gene expression (ddCt) method. All the measurements were performed in triplicate. The sequences of the primer pairs were as follows: (MFAP5 F: GCATCGGCCGGTTAAACAAT, R: TCACAGGGAGGAAGTCGGAA) Hes1 (F: GTGTCAACACGACACCGGAT, R: GGAATGCCGCGAGCTATCTT), Hes5 (F: AGTCCCTGCCGTTTTAGGAC, R: GAGCCCCGGCACTACAAATA), slug (F: TCTGGGCTGGCCAAACATAA, R: TTCTCCCCCGTGTGAGTTCTA), Snail (F: CGAGTGGTTCTTCTGCGCTA, R: CTGCTGGAAGGTAAACTCTGGA), Zeb1 (F: ACCTGCCAACAGACCAGACA, R: TCTTGCCCTTCCTTTCCTGTGT), Zeb2 (F: AGTGTGCCCAACCATGAGTC, R: ACTGGACCATCTACAGAGGC), Twist1 (F: GGCCAGGTACATCGACTTCC, R: CATCCTCCAGACCGAGAAGG).

### Immunofluorescence analysis

Cells were cultured on glass coverslips in a 24-well plate, fixed with 4% paraformaldehyde, permeabilized using 0.1% Triton X-100, washed with 1× PBS, and blocked with 5% bovine serum albumin. The cells were incubated with MAGP2 primary antibody (1:100 dilution, Abcam), followed by incubation with a Goat Anti-rabbit IgG/RBITC antibody (1:100 dilution, Bioss). Coverslips were mounted with vectashield mounting medium containing diamidino-2-phenylindole (DAPI, sigma) nuclear stain and examined under an IX51 fluorescence microscope (Olympus), then the images were constructed using software.

### In vitro migration and invasion assay

#### Cell proliferation assay

Cell proliferation was monitored by the colorimetric water-soluble tetrazolium salt (CCK8) assay using a Cell Counting Kit-8 (DOJINDO) according to the manufacturer’s instructions. Breast cancer cells were seeded onto 96-well plates (3 × 10^3^ cells/well). Added CCK8 reagent (10 µl/well) at 0, 24, 48 or 72 h, respectively. Cells were incubated for another 3 h at 37 °C. The number of viable cells was assessed by measurement of the absorbance at 450 nm using a RT-2100C microplate reader (Rayto).

#### Cell-fibronectin adhesion assay

The 96-well plates were coated with 10 µg/ml of fibronectin (sclencell). Breast cancer cells (3 × 10^3^ cells/well) were placed at 37 °C for 30 min, and then rinsed with PBS. The cells were digested and suspended with non-serum medium. Later on, the cells were seeded onto the above 96-well plate at a 90% density. After adhesion for 30 min at 37 °C, the cells were washed with PBS for 2 times and then visualized under a microscope. To minimize the bias, at least three randomly selected fields were quantified using 100× magnification, and the average number of cells was taken.

#### In vitro scratch assay

Draw two horizontal lines in the back of the 96-well plate across the hole, plate growing cells at 90% confluence to form a monolayer. Scrape the cell monolayer in a straight line to create a ‘‘scratch’’ with a p200 pipet tip. Remove the debris and smooth the edge of the scratch by washing the cells once with PBS and then replace with serum-free medium. Place the plate under a phase-contrast microscope and acquire the 0 h images of the scratch. The cells continued to be cultured in the incubator at 37 °C for 24 h. After the incubation, the plate was placed under a phase-contrast microscope, the reference point was matched, and the photographed region acquired images were aligned. The cell migration distance in the same field of views was analyzed and the effect of MFAP5 on cell migration ability was compared.

#### Transwell migration assay

BT-20 cells were cultured in serum-free medium for 24 h, digested and suspended in 100 μl serum-free medium. The cell density was adjusted t to 5 × 10^5^/ml and they were seeded into the upper chamber of a transwell inserted with an 8-mm pore size membrane (Corning). The culture medium chemoattractant of the upper and down transwell chamber was adjusted according to the experimental needs. After incubation for 24 h at 37 °C, non-migrated cells in the upper chamber of the transwell insert were removed with a cotton swab, and the migrated cells on the underside of the filter membrane were fixed and stained with 0.1% crystal violet. The number of migrated cells was counted in five randomly selected microscopic fields and photographed. The experiments were independently repeated thrice.

#### In vitro angiogenesis assay

The matrigel (Yeasen) solution was applied to 96-well plate at 50 μl per well. The matrix gel was placed on ice for 20 min, then incubated at 37 °C for 24 h. Cells (BT20 mock, BT20-LV-vehicle, BT20-LV-MFAP5, HS578T mock, HS578T-control-shRNA and HS578T-MFAP5-shRNA) were cultured at 50% density in free-serum medium for 48 h, and then cell supernatant was collected, respectively. HUVEC cells were suspended with 100 μl the above cell supernatant and added to the matrix-coated 96-well plate. Positive control (complete culture with 10% FBS) and negative control (serum-free basal medium) group were established and continued to be cultured for 6 h. Each well was placed under inverted microscope, and pictures were taken to observe the angiogenesis and analysis the average length of blood vessels.

### Western blotting

Cells were harvested from cultured dishes and were lysed for 30 min in cold lysis buffer. Cell extracts were collected and centrifuged at 13,000 rpm for 15 min. Total proteins from whole cell lysates were boiled for 10 min in 1× SDS buffer, resolved by 12% SDS-PAGE, and then electrotransferred to nitrocellulose membranes. After the electrophoretic transfer, the membranes were blocked with 5% non-fat milk in Tris-buffered saline. After blocking the membranes were incubated overnight at 4 °C with primary antibodies at recommended concentration. The antibodies for Smad2/Smad3, phospho-Smad2/Smad3, E-cadherin, N-cadherin, vimentin and Type I collagen were purchased from Abcam Technologies. Then, the membranes were incubated with horseradish peroxidase-conjugated secondary antibodies (anti-rabbit or anti-mouse IgG) (Abcam). Signals were detected with immobile Western chemiluminescent HRP Substrate (Millipore). β-actin (Abcam) was served as the loading control.

### Tumorigenicity assays

All animal experiments were carried out under a license from the Institutional Animal Care and Use Committee at Second Military Medical University. For tumorigenicity and metastasis of human breast cancer in vivo, 5 × 10^6^ of BT20-LV-vehicle and BT20-LV-MFAP5 cells which suspended in PBS were injected into the 4 weeks old nude immunocompromised mice (Shanghai Super-B&K Laboratory). In experiment 1, BT20-LV-vehicle and BT20-LV-MFAP5 cells were injected into the mammary fat pad of nude mice respectively. In experiment 2, the nude mice were transduced with BT20-LV-vehicle or BT20-LV-MFAP5 cells by tail intravenous injection. From the 2nd week after tumor cell inoculation, tumor size was monitored once a week and determined with caliper. Four to eight weeks from the onset of the study, the mice were photographed and anesthetized; the tumors were removed and weighted.

In order to analyze the metastasis of tumors, the left axillary lymph nodes of mice in experiment 1 were taken to evaluate the tumor metastatic focus by IHC and RT-PCR assays. Mice in experiment 2, lungs were dissected to analyze metastases situation.

### Immunohistochemistry (IHC)

The mouse tumor tissues were fixed in 10% neutralized formalin and embedded in paraffin blocks. Sections (5 µm) were then prepared for immunohistochemical examination. After deparaffinization and rehydration, antigen retrieval was performed by boiling in 0.01 mmol/L citrate buffer (pH 6.0) for 15 min. After inhibition of endogenous peroxidase activity for 30 min with methanol containing 3% H_2_O_2_. The sections were blocked with 2% bovine serum albumin in PBS for 30 min and incubated with primary antibody overnight at 4 °C. Staining with secondary antibodies (EnVision) was then performed before development using DAB substrate (Dako) according to the manufacturer’s procedure. The cytoplasm was counterstained with hematoxylin.

### RNA sequencing and data analysis

Total RNA samples were obtained to generate RNA libraries for each sample. After the samples were qualified, mRNA was fragmented by heating. These short mRNA and random hexamers were used to generate the first cDNA and then the second cDNA was synthesized. The second cDNA was purified using VAHTSTM DNA Clean Beads (Vazyme) and then ligated to sequencing adapters. The fragments were amplified by using polymerase chain reaction (PCR) and purified using VAHTSTM DNA Clean Beads and then sequenced using an Illumina HiSeq (Vazyme). Raw sequence data were assessed, and sequences containing adaptor tags and those of low quality were excluded. DESeq [26] was used to normalize RNA-seq fragment counts to compare the relative abundances of transcripts, and nbinomTest method was used to test for differential expression between cases and controls. Only differentially expressed genes with *P*-value < 0.05 and an absolute log2 fold-change > 2 were selected for differentially expressed genes. The differentially expressed genes were also used for Gene Ontology (GO) and Kyoto Encyclopedia of Genes and Genomes (KEGG) analysis [27].

### Statistical analysis

Results were expressed as mean ± SD. For comparisons between two different groups, statistical significance was determined using the Student’s t-test. A Kruskal–Wallis test followed by Dunn’s post hoc test and one-way ANOVA followed by Bonferroni post hoc test were used to determine non-parametric data and continuous variables, respectively. Data were analyzed with GraphPad Prism. In all statistical tests, *P* < 0.05 was considered statistically significant.

## Results

### MFAP5 expression levels in human breast cancer cell lines

In order to choose the proper BLBC cell lines, the MFAP5 level in human breast cancer cell lines BT20 and HS578T was analyzed. Quantitative real-time PCR demonstrated HS578T cells had higher level of MFAP5 than BT20 cells (Additional file 1: Table S1). Finally, we selected HS578T (higher expression of MFAP5) and BT20 (lower expression of MFAP5) for the following studies.

### MFAP5 promoted cancer cell proliferation, adhesion, migration and invasion in vitro

To determine the role of MFAP5 in BLBC, we transfected BT20 cells with MFAP5 lentivirus to obtain stable cell line BT20-LV-MFAP5 with MFAP5 overexpression. Besides, to inhibit the expression of MFAP5, HS578T cells were transfected with MFAP5 shRNA. The results of qRT-PCR confirmed that MFAP5 was overexpressed in BT20-LV-MFAP5 cells and downregulated in HS578T-MFAP5-shRNA cells (Additional file 2: Figure S1).

**Fig. 1:**
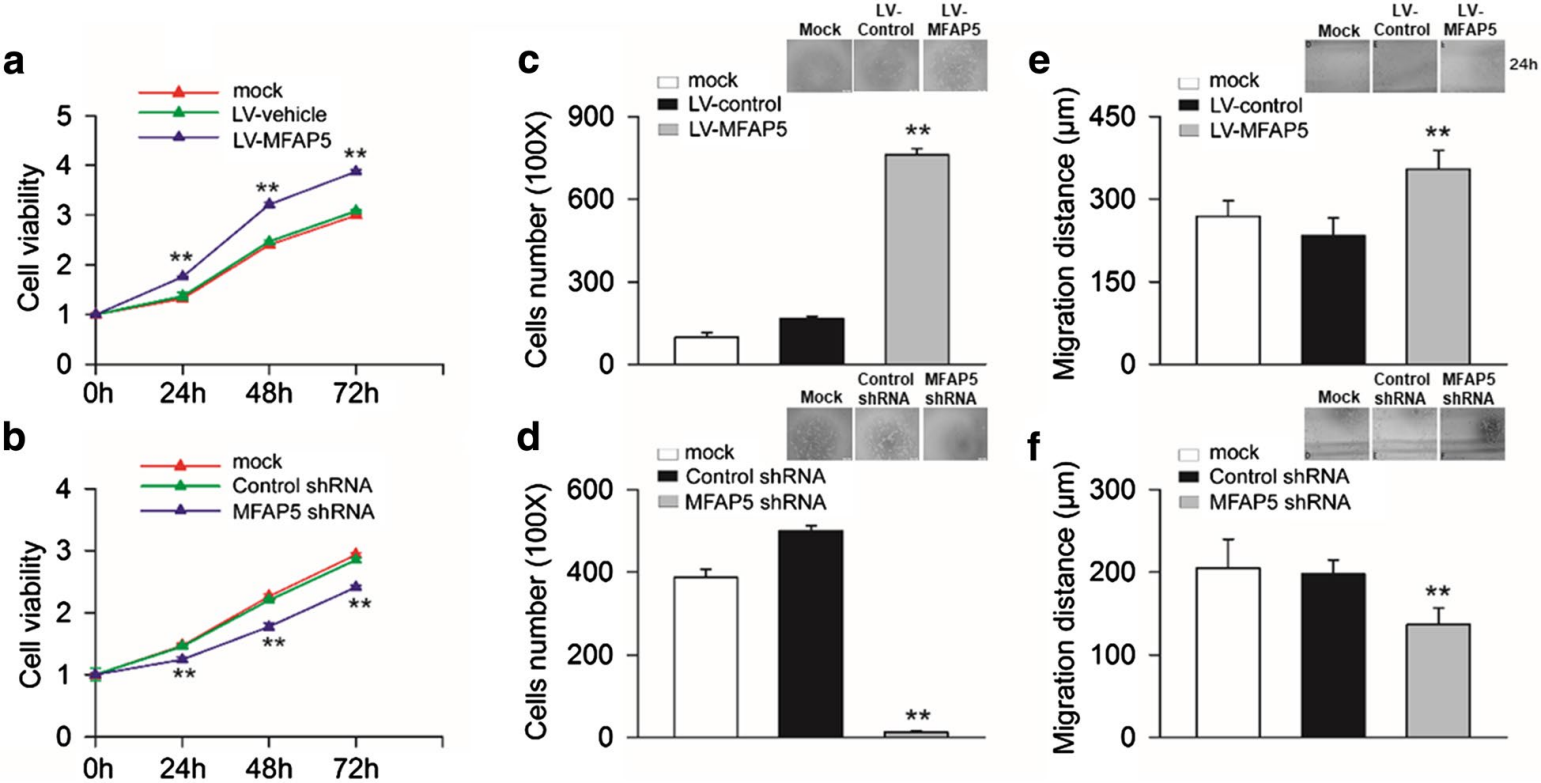
MFAP5 regulated the proliferation, adhesion and migration of BT20 and HS578T cells. BT20 cells in mock, LV-vehicle, LV-MFAP5 groups and HS578T cells in mock, control shRNA, MFAP5 shRNA groups were cultured at different time as indicated and then the proliferation, adhesion and migration of cells were examined. **a** Overexpressed MFAP5 time-dependently increased the proliferation of BT20 cells. **P < 0.01 vs LV-vehicle. **b** Knockdown MFAP5 in HS578T cells by shRNA inhibited cell proliferation. **P < 0.01 vs control shRNA. **c** Compared with LV-vehicle group, overexpressing MFAP5 significantly promoted BT20 cells adhesion. **P < 0.01 vs LV-vehicle. **d** Knockdown MFAP5 in HS578T cells by shRNA inhibited cell adhesion. **P < 0.01 vs control shRNA. **e** The migration of BT20 cells in LV-MFAP5 group was increased compared with those in LV-vehicle group. **P < 0.01 vs LV-vehicle. **f** Knockdown MFAP5 in HS578T cells by shRNA inhibited cell motility compared with those in control shRNA group. **P < 0.01 vs control shRNA

Next, we examined the function of MFAP5 in breast cancer cells. CCK8 assay revealed that BT20 cells transfected with MFAP5 lentivirus grew more rapidly than those transfected with vehicle (Fig. 1a). In contrast, compared with control shRNA group, cells in HS578T-MFAP5-shRNA group showed decreased cell viability (Fig. 1b). These data indicated that MFAP5 promoted BLBC growth in vitro. The cell-fibronectin adhesion assay revealed that overexpression of MFAP5 stimulated the adhesion of BT20 cells (Fig. 1c). When the MFAP5 gene was knocked down in HS578T cells, the adhesive behavior was decreased significantly (Fig. 1d). Next, we further assessed the effects of MFAP5 on cell migration, a key determinant of malignant progression and metastasis. As shown in Fig. 1e, overexpression of MFAP5 significantly increased BT-20 cells migration, while knockdown of MFAP5 in HS578T cells, the scratch wound-healing motility were decreased markedly (Fig. 1f). These results indicated MFAP5 promoted cell motility.

**Fig. 2:**
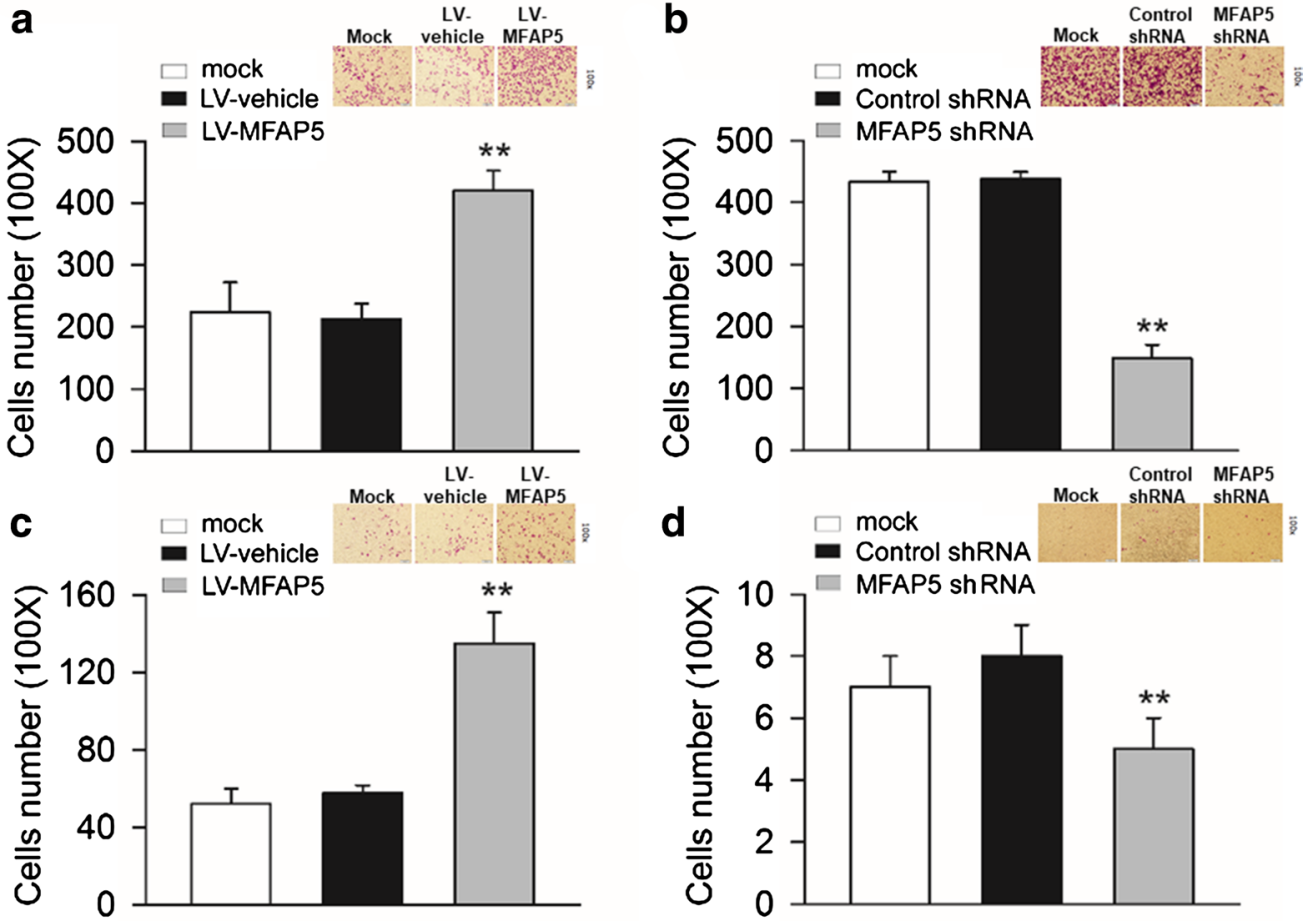
MFAP5 regulates the invasion of BT20 and HS578T cells. Cell invasion was measured by transwell migration assay. **a** Compared to BT20-LV-vehicle cells, the invasion was increased of BT20-LV-MFAP5 cells. **P < 0.01 vs LV-vehicle. **b** The invasion of HS578T cells in MFAP5 shRNA group was decreased compared with those in control shRNA group. **P < 0.01 vs control shRNA. **c** The supernatant of BT20-LV-MFAP5 cells promoted the invasion of BT20 cells. **P < 0.01 vs LV-vehicle. **d** HS578T cell invasion was inhibited by the supernatant of HS578T-MFAP5-shRNA cells. **P < 0.01 vs control shRNA

In addition, invasive rate in breast cells was detected using a transwell assay. As shown in Fig. 2a, b, overexpression of MFAP5 promoted BT-20 cell invasion, while the ability of cell invasion was significantly weakened by MFAP5 knockdown in HS578T cells compared with those in control shRNA group. We also discovered the supernatant of BT20-LV-MFAP5 cells could promote the invasion of BT20 cells (Fig. 2c). Similarly, the invasion of HS578T cells was decreased by the supernatant of HS578T cells transfected with MFAP5 shRNA (Fig. 2d). These results proposed a functional role for MFAP5 in mediating cell migration and invasion in BLBC cells in vitro.

### MFAP5 induced angiogenesis of HUVEC cells

**Fig. 3:**
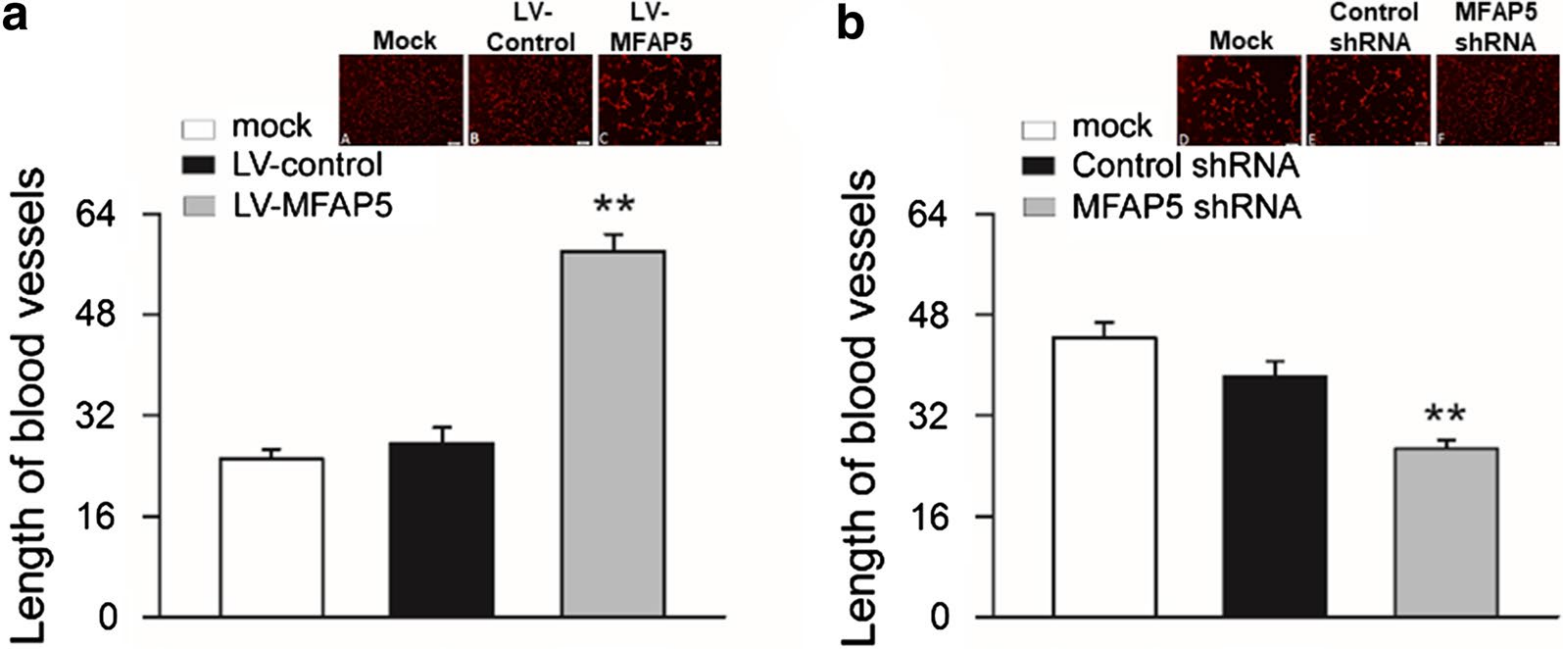
MFAP5 regulated angiogenesis of HUVEC cells. **a** The length of blood vessels of HUVEC cells in the supernatant of BT20-LV-MFAP5 cells was increased compared with those in BT20-LV-vehicle cell supernatant. **P < 0.01 vs LV-vehicle. **b** Compared to HUVEC cells in the supernatant of HS578T-control-shRNA cells, length of blood vessels was decreased in the supernatant of HS578T-MFASP5-shRNA cells. **P < 0.01 vs control shRNA

To explore the role of MFAP5 on angiogenesis, we cultured HUVEC cells with the supernatant of BT20-LV-vehicle and BT20-LV-MFAP5 cells and found the average length of the HUVEC blood vessels in supernatant of BT20-LV-MFAP5 group was about three times of that in BT20-LV-vehicle group (Fig. 3a). In contrast, comparing with HUVEC cells in the supernatant of HS578T-control-shRNA cells, the average length of the blood vessels decreased in HS578T-MFAP5-shRNA cells supernatant (Fig. 3b). These results showed that MFAP5 could promote the angiogenesis of HUVEC cells.

### MFAP5 promoted BLBC tumor growth and metastasis in vivo

**Fig. 4:**
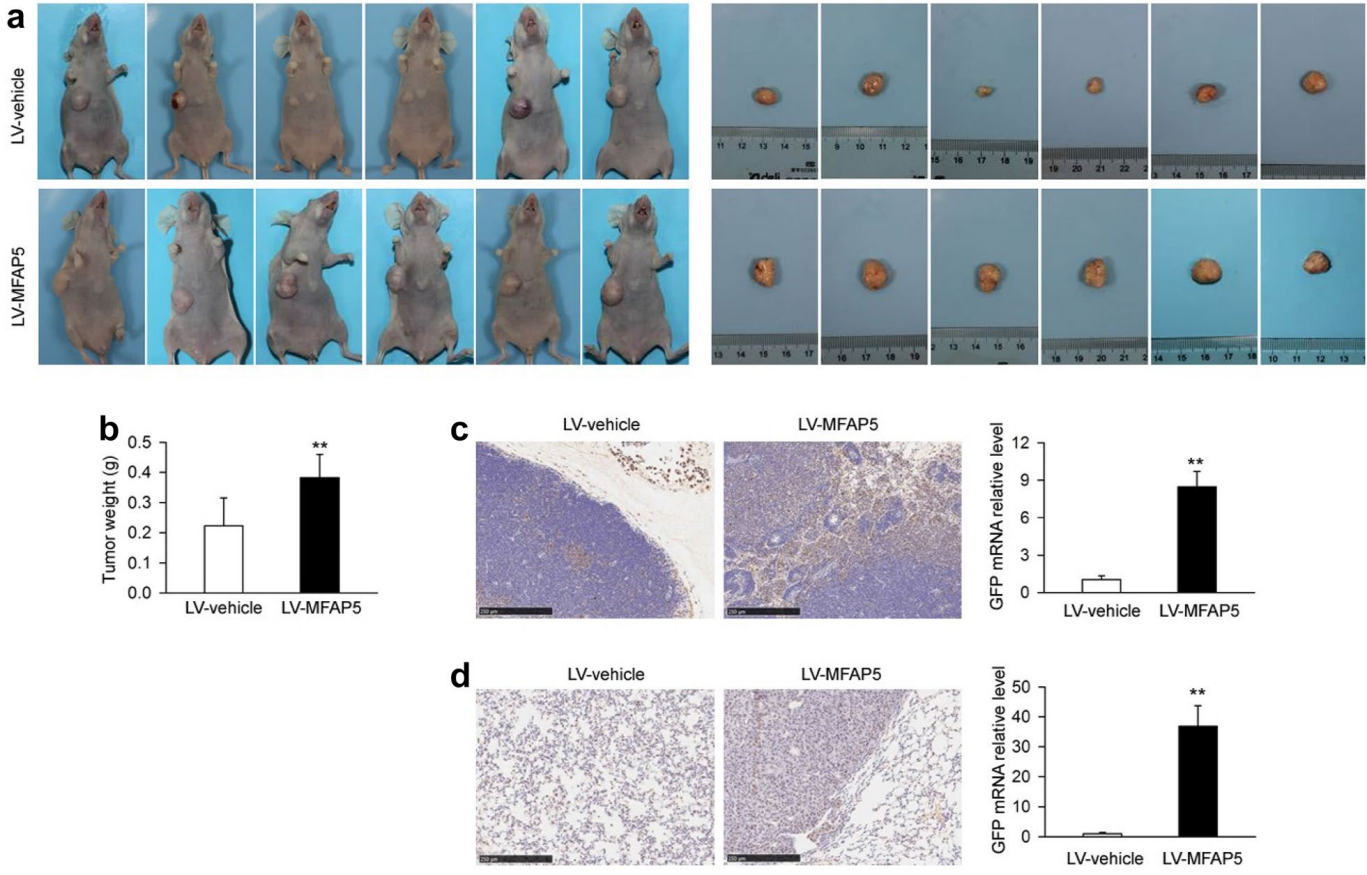
MFAP5 regulated tumor proliferation and BT20 cell metastasis. **a** The volume of tumors in BT20-LV-MFAP5 nude mice was bigger than those in BT20-LV-vehicle group. **b** Compared with nude mice in BT20-LV-vehicle group, the weight of tumors in BT20-LV-MFAP5 group was significantly increased. **P < 0.01 vs LV-vehicle. **c** IHC and RT-PCR showed that the BT20 cells transferred to axillary lymph nodes in BT20-LV-MFAP5 nude mice were enhanced compared with these in BT20-LV-vehicle group. **P < 0.01 vs LV-vehicle. **d** IHC and RT-PCR demonstrated the lung metastasis of BT20 cells was increased in BT20-LV-MFAP5 nude mice. **P < 0.01 vs LV-vehicle

To determine the effect of MFAP5 on BLBC tumor growth in vivo, stable BT20-LV-MFAP5 cells with modified MFAP5 expression and normal control BT20-LV-vehicle cells were respectively injected into two groups of nude mice. In experiment 1, the volume of tumors in BT20-LV-MFAP5 nude mice was much bigger than that in BT20-LV-vehicle group (Fig. 4a). Besides, the weight of tumors in BT20-LV-MFAP5 nude mice (0.38 ± 0.08 g) was significantly increased compared with that in BT20-LV-vehicle group (0.22 ± 0.09 g) (Fig. 4b). IHC showed axillary lymph nodes metastasis of BT20-LV-MFAP5 group was more serious than BT20-LV-vehicle cells. qRT-PCR results further demonstrated that the GFP mRNA expression of axillary lymph nodes in BT20-LV-MFAP5 group (8.47 ± 1.23) was about 8 times higher than that in BT20-LV-vehicle group (1.04 ± 0.31) (Fig. 4c). In addition, lung metastasis was also found in BT20-LV-MFAP5 group in experiment 2 (Fig. 4d). The GFP mRNA expression of lung in BT20-LV-MFAP5 group was 36.9 ± 6.78 compared with 1.06 ± 0.34 of lung in BT20-LV-vehicle group (Fig. 4d). These results suggested that MFAP5 promoted the tumorigenesis and metastasis of BLBC in vivo.

### MFAP5 activated TGF-β and Notch pathways

**Fig. 5:**
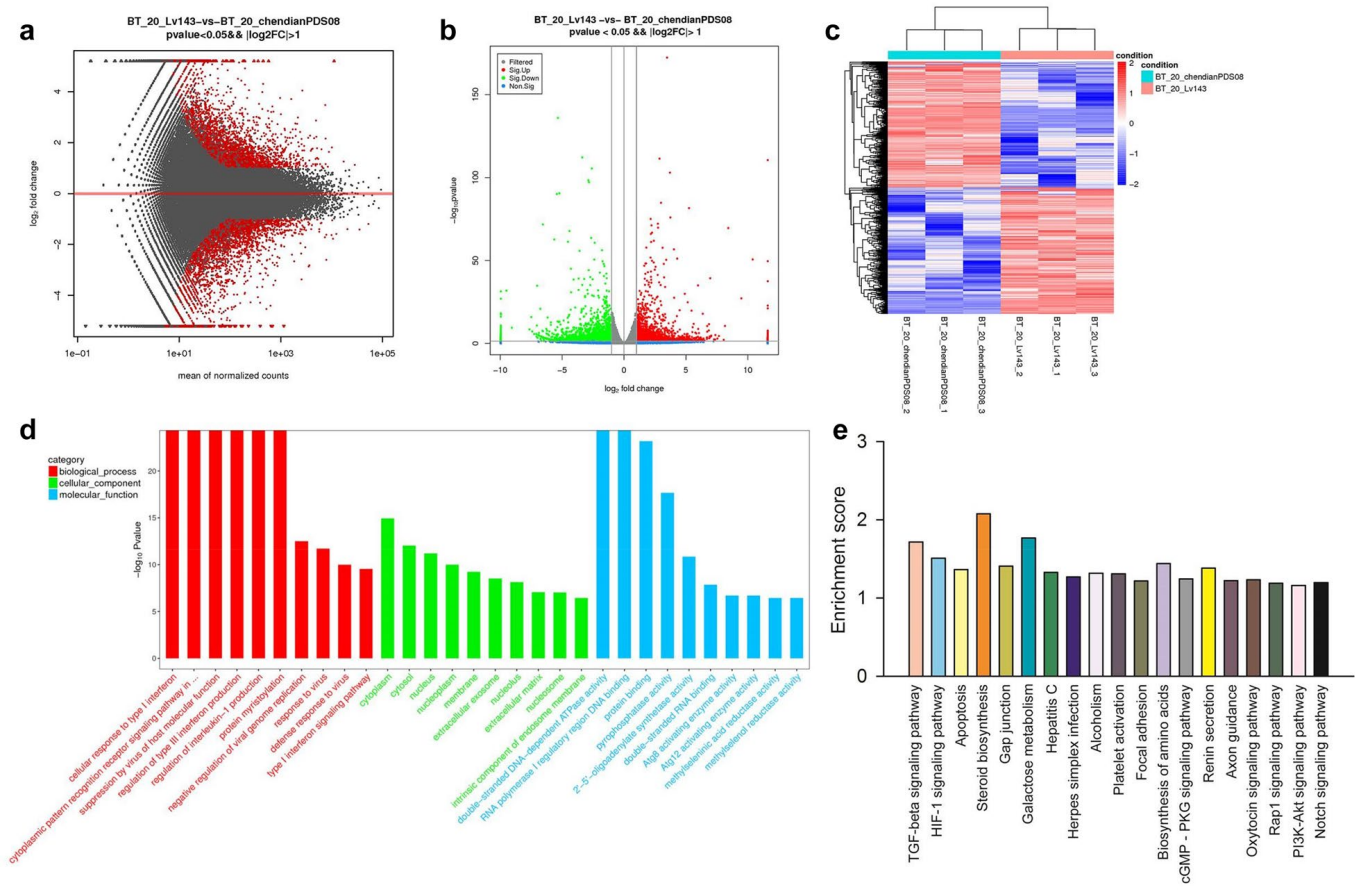
GO/KEGG pathway enrichment analysis of differentially expressed genes between BT20-LV-vehicle and BT20-LV-MFAP5 cells using RNA-sequencing. **a** M (log ratio) and A (mean average) scales indicated the mRNA differentially expressed (red spots) in BT20-LV-vehicle and BT20-LV-MFAP5 cells. **b** The mRNA differentially expressed volcano plots in BT20-LV-vehicle and BT20-LV-MFAP5 cells. **c** Heatmap of differential expression levels of mRNA (log2RPKM) in these two cells. **d** Differentially expressed genes enriched and identified by GO analysis in these two cells. **e** Differentially expressed mRNA in 19 pathways including TGF-β and Notch pathway were figured out by KEGG analysis

**Fig. 6:**
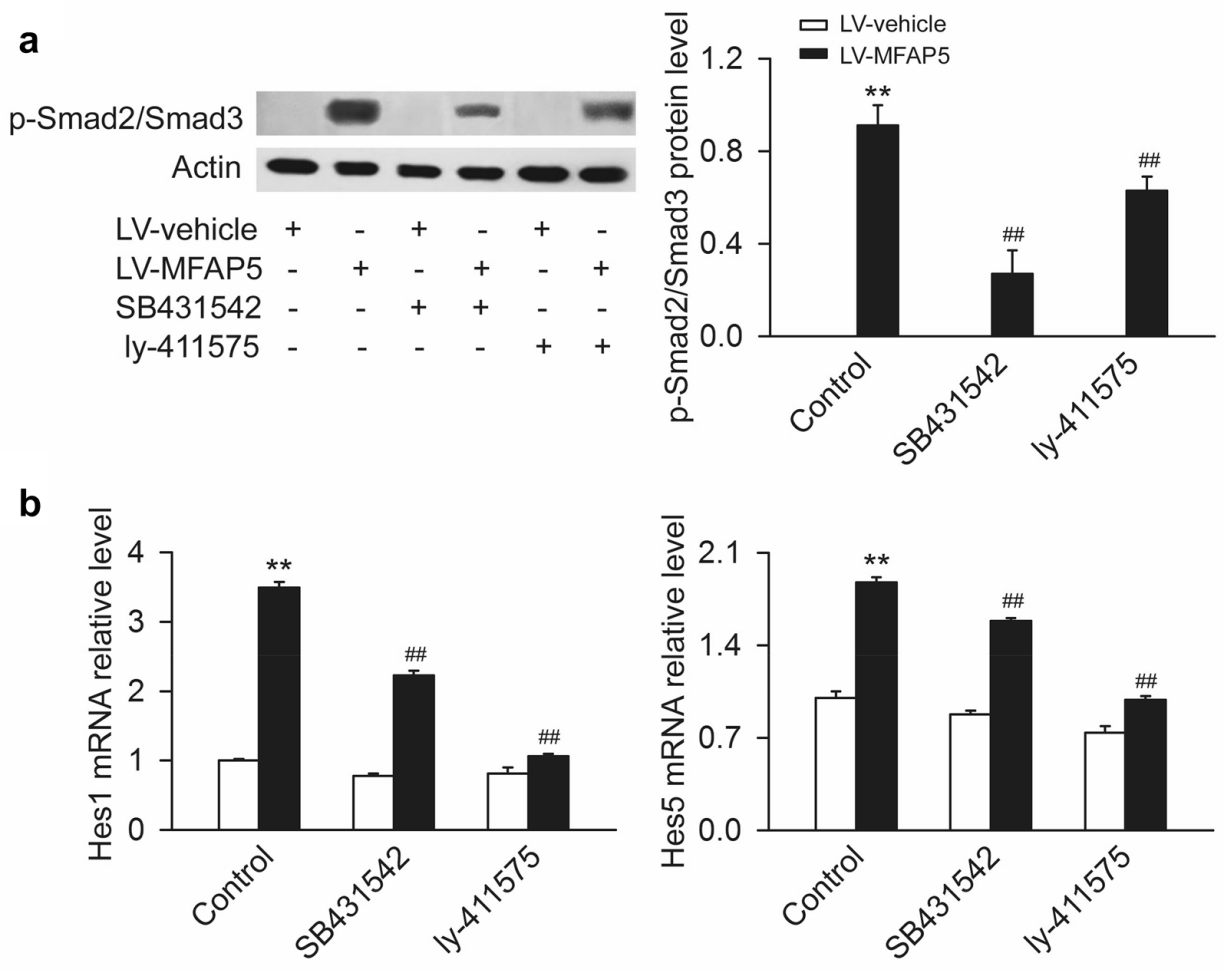
MFAP5 regulated the activation of TGF-β and Notch pathways in BT20 cells. BT20-LV-vehicle and BT20-LV-MFAP5 cells were treated with SB431542 or ly-411575 as indicated for 24 h and then collected to detect the protein level of p-Smad2/Smad3 by western blot and the mRNA levels of Hes1 and Hes5 by RT-PCR. **a**, **b** Compared with LV-vehicle group, overexpression of MFAP5 significantly improved the overexpression of p-Smad2/Smad3 and the mRNA levels of Hes1, Hes5 which were strikingly attenuated by SB431542 and ly-411575. **P < 0.01 vs LV-vehicle control; ^##^*P* < 0.01 vs LV-MFAP5 control

The mRNA differentially expressed between BT20-LV-vehicle and BT20-LV-MFAP5 cells were presented as M (log ratio) and A (mean average) scales plots or volcano plots (Fig. 5a, b). The mRNA differentially expression levels (log2RPKM) in these two cells were shown in Heatmap (Fig. 5c). “There were 4138 significantly differentially expressed genes (P < 0.05) between BT20-LV-vehicle and BT20-LV-MFAP5 cells.” In addition, GO/KEGG pathway enrichment analysis on these genes identified 19 significant (P < 0.05) pathways including TGF-β and Notch pathways which were mediated in the activation of EMT (Fig. 5d, e). This result suggested that MFAP5 may play an important role in the activation of TGF-β and Notch pathways. In order to prove this hypothesis, we examined the levels of Smad2/Smad3 (a vital signal in TGF-β pathway) and two factors regulated by Notch pathway Hes1, Hes5 in BT20-LV-vehicle and BT20-LV-MFAP5 cells. Selected concentration of TGF-β receptor inhibitor SB431542 (10 μM) and Notch receptor inhibitor ly-411575 (0.1 μM) were used for subsequent experiments (Additional file 3: Figure S2). As shown in Fig. 6a, Table 1, overexpression of MFAP5 promoted the phosphorylation of Smad2/Smad3, which was attenuated by TGF-β or Notch inhibitor, and the degree of reduction by TGF-β inhibitor group was more obvious than that in Notch inhibitor group. The qRT-PCR results showed that Hes1 and Hes5 were upregulated by overexpression of MFAP5 in the cells. Meanwhile, the levels of Hes1 and Hes5 were down-regulated after treated with inhibitors, and Notch inhibitor exerted a better effect than TGF-β inhibitor (Fig. 6b). These results suggested that MFAP5 may promote the activation of both TGF-β and Notch signaling pathways.

**Table 1:** Relative concentration of Smad2/3 expression in western blot test

	Sample	NC	MEAP5	NC + SB431542	MFAP5 + SB431542	NC + ly411575	MFAP5 + ly411575
p-Smad2/Smad3	Gray value	0	302,830	0	126,610	0	206,209
		0	246,130	0	76,568	0	171,906
		0	292,443	0	79,480.3	0	186,458
	Average	0	280,468	0	94,219	0	188,191
	Gray value of each molecule relative to beta-actin (G1)	0.00	0.91	0.00	0.28	0.00	0.63
	Compared with control group	0.00	0.10	0.00	0.08	0.00	0.06
	Compared with control group (G1/NC)	0.00	9.29	0.00	2.89	0.00	6.51
Internal reference	Gray value	365,609	302,497	300,448	314,709	340,436	326,191
		303,144	316,665	357,508	336,392	292,601	284,270
		335,737	309,839	352,063	352,295	287,281	279,450
	Average	334,830	309,667	336,673	334,465	306,773	296,637
	Compared with control group	0.09	0.02	0.09	0.06	0.10	0.09

### MPAP5 regulated epithelial–mesenchymal transition and motility of BLBC cells through TGF-β and Notch pathways

**Fig. 7:**
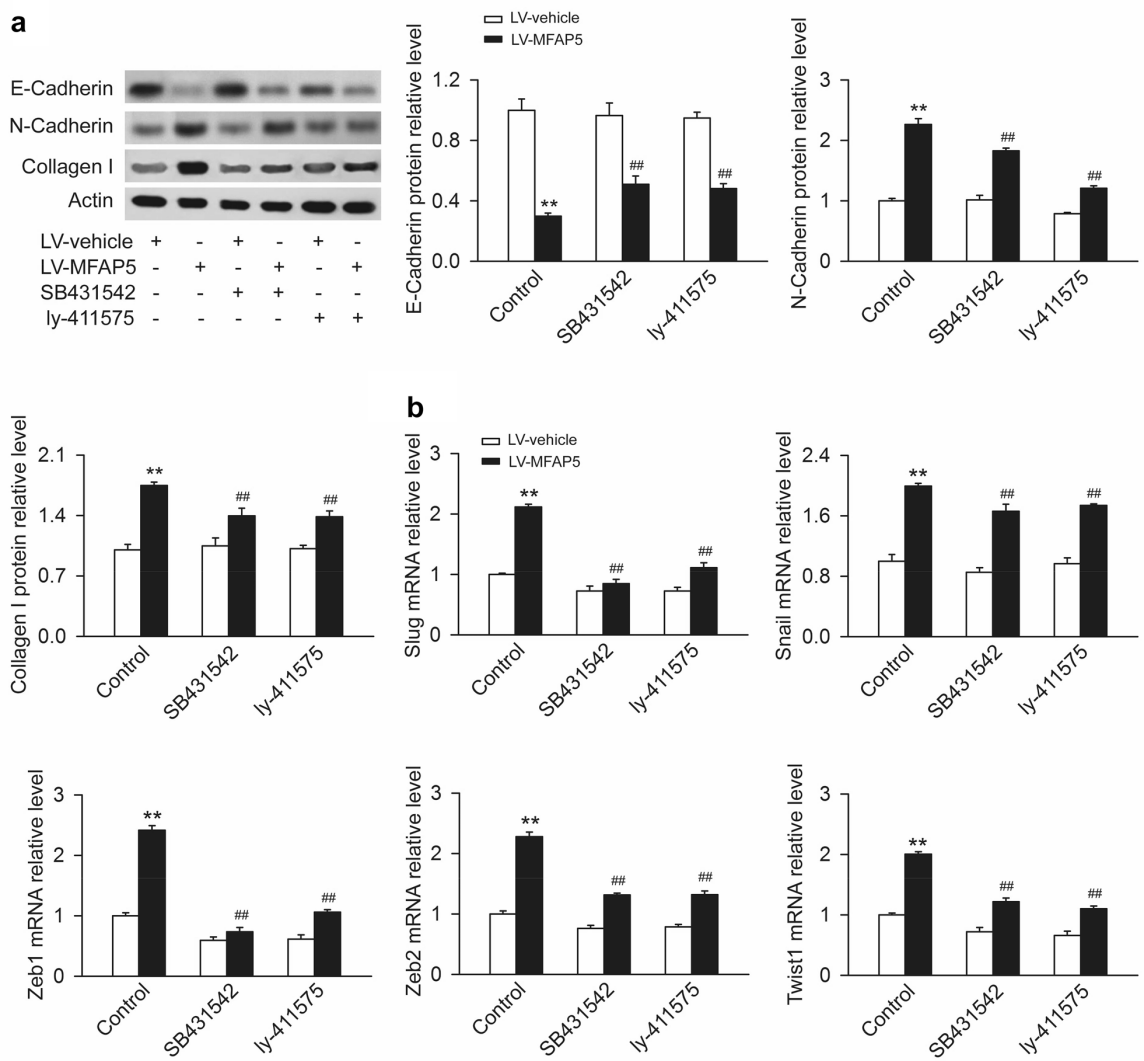
MPAP5 regulated epithelial–mesenchymal transition of BLBC cells through TGF-β and Notch pathways. BT20-LV-vehicle and BT20-LV-MFAP5 cells were treated with SB431542 or ly-411575 as indicated for 24 h and then collected to test the protein levels of E-cadherin, N-cadherin and Type I Collagen by Western blotting and the mRNA levels of Slug, Snail, Zeb1, Zeb2 and Twist by RT-PCR. **a** Overexpression of MFAP5 reduced the protein level of E-cadherin and increased N-cadherin and Collagen I expression in BT20 cells. SB431542 and ly-411575 partly abolished the inhibitory effect of MFAP5 on E-cadherin and the promoting role on N-cadherin and collagen I. **P < 0.01 vs LV-vehicle control; ^##^*P* < 0.01 vs LV-MFAP5 control. **b** Compared with LV-vehicle group, the mRNA levels of Slug, Snail, Zeb1, Zeb2 and Twist were significantly increased in BT20 cells of LV-MFAP5 group which were decreased by SB431542 and ly-411575. **P < 0.01 vs LV-vehicle control; ^##^P < 0.01 vs LV-MFAP5 control

EMT is a process that epithelial cells transform into mesenchymal cells by losing cellular morphology and migratory capacity [28]. We then assessed the role of MFAP5 in EMT and examined whether the TGF-β and Notch signaling pathways mediated MFAP5-induced EMT and metastasis. We tested the protein levels of the epithelial marker E-cadherin, N-cadherin and the mesenchymal marker Type I Collagen of cells in BT20-LV-vehicle and BT20-LV-MFAP5 groups. The results indicated MFAP5 overexpression increased levels of N-cadherin, Type I collagen as well as decreased E-cadherin in the cells, which were dramatically revered by TGF-β or Notch inhibitor (Fig. 7a). In EMT programming, pleiotropic EMT transcription factors form an interaction network and act in concert to regulate the EMT phenotype. MFAP5 markedly raised the mRNA levels of EMT transcription factors include Slug, Snail, Zeb1, Zeb2 and Twist which were largely decreased by SB431542 and ly-411575 (Fig. 7b). These results revealed that MFAP5 promoted EMT programming in BLBC cells and TGF-β/Notch signaling pathways, at least in part.

**Fig. 8:**
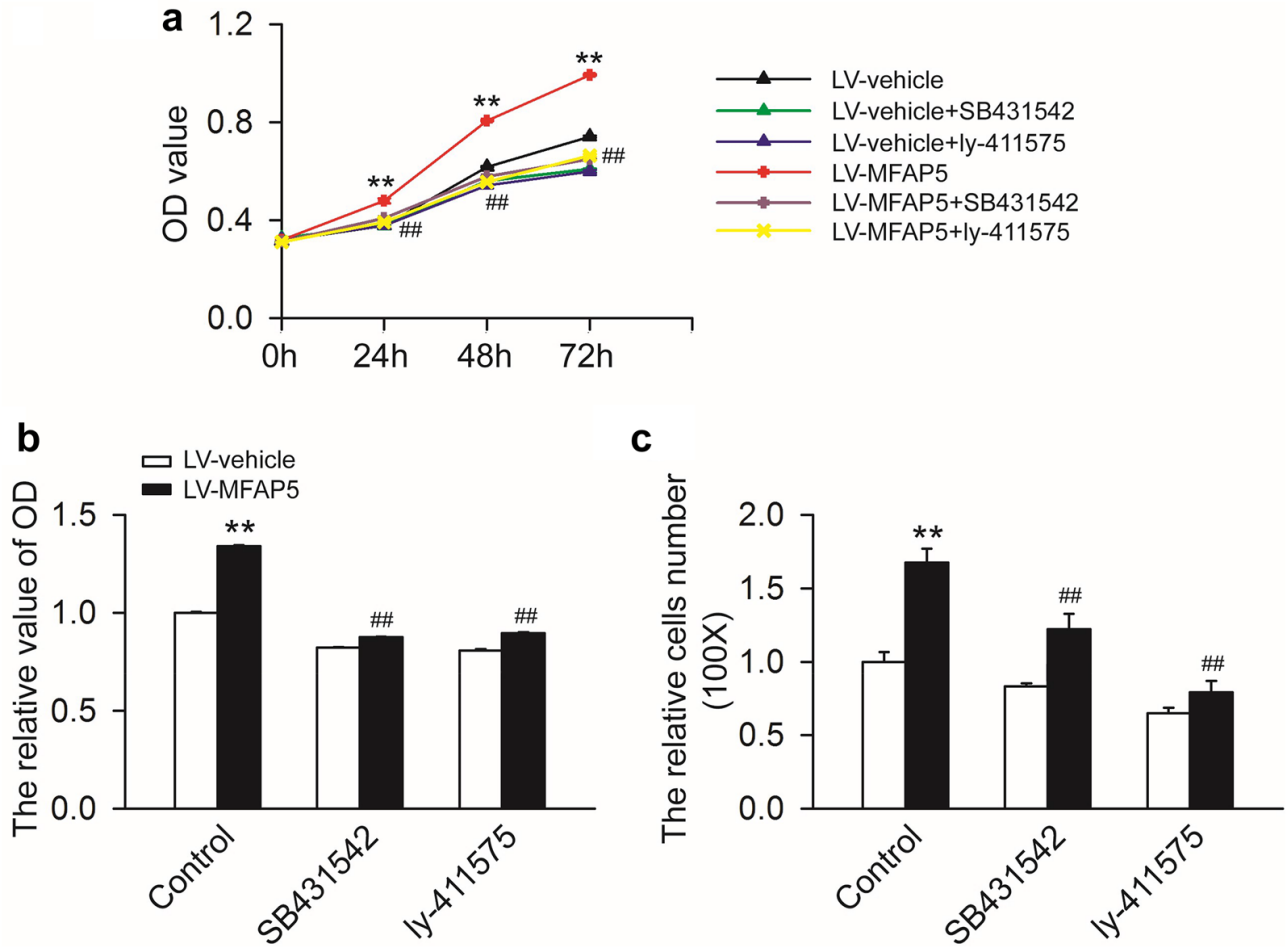
TGF-β and Notch pathways mediated the promoting effects of MPAP5 on BLBC cells motility. **a** BT20-LV-vehicle and BT20-LV-MFAP5 cells were treated with SB431542 or ly-411575 as indicated for 0, 24, 48 and 72 h. Then the OD value of cells was measured. Overexpression of MFAP5 time dependently improved the OD value compared to LV-vehicle group. But OD value elevated by MFAP5 was strikingly weakened by SB431542 and ly-411575. **P < 0.01 vs LV-vehicle control; ^##^*P* < 0.01 vs LV-MFAP5 control. **b** The OD value of cells at 72 h was presented. MFAP5 significantly increased cell OD value which was attenuated by SB431542 and ly-411575. **P < 0.01 vs LV-vehicle control; ^##^P < 0.01 vs LV-MFAP5 control. **c** BT20-LV-vehicle and BT20-LV-MFAP5 cells were treated with SB431542 or ly-411575 as indicated for 24 h, and the invasion was measured by transwell migration assay. Compared with LV-vehicle group, the number of BT20 cell invasion was significantly increased in LV-MFAP5 group which were remarkably abolished by SB431542 and ly-411575. **P < 0.01 vs LV-vehicle control; ^##^*P* < 0.01 vs LV-MFAP5 control

Next, we examined whether TGF-β and Notch signaling pathways were responsible for changes in cell motility regulated by MFAP5. We evaluated the influence of TGF-β or Notch inhibitor on the biological behavior of cells by the CCK-8 and transwell assays. As illustrated in Fig. 8, the promotion effects of MFAP5 on BT20 cell proliferation and migration were partly abolished by TFG-β or Notch inhibitor. These data suggested that MFAP5 might promote BLBC aggressive cellular traits via TGF-β/Notch pathways.

## Discussion

To date, the genetic profile and biologic basis of BLBC are poorly understood. Currently there is no targeted treatment measure for breast cancer. Therefore, more and more attention has been paid to the etiology, pathogenesis, especially invasion and metastasis of breast cancer. With the further research on the molecular mechanism of tumor cell growth, proliferation and apoptosis, it is an urgent task for breast cancer to find the biological marker of its targeted therapy. Activation of EMT is thought to initiate this early step in the metastatic cascade [29]. Notably, EMT has been demonstrated to be the most dominant pattern of intrinsic gene expression in BLBC [12]. Some studies have implicated that the development and progression of BLBC involves several signaling pathways such as TGF-β [14], MEK/PI3K [23, 30], integrin [31], Notch pathways [32], and NF-κB [33]. It is reported that MFAP5 is associated with microfibrils and modulates endothelial cell behavior [18, 20]. MFAP5 also promotes angiogenesis and interacts with Notch1 by either activating or suppressing its activity, depending on the cell type involved [21, 34]. In our previous study, we demonstrated that mRNA expression of MFAP5 was highly upregulated in BLBC [35]. In this study, we found that MFAP5 participated in carcinogenesis, development and progression of BLBC. The upregulation MFAP5 in BLBC was related to TNM staging and axillary lymph node metastasis. Up to date, no data show that expression of MFAP5 is associated with BLBC. This study contributes to the understanding of the molecular mechanism by which MFAP5 overexpression in BLBC promotes tumor progression. The results of this study suggest that MFAP5 may activate the TGF-β and Notch signaling pathways to promote EMT, leading to an aggressive phenotype and tumor progression to metastasis.

Through the study of ovarian serous papillary adenocarcinoma, Mok et al. [22] showed that MFAP5 can be used as an independent predictor of poor prognosis in patients. It plays a very important role in the integration of elastic microfibrils and the regulation of endothelial cell behavior and cell survival. Previous studies have shown that MAGP2 (MFAF5) promotes tumor and endothelial cell survival and endothelial cell motility through α_V_β_3_ integrin-mediated signaling [20]. Results of this study revealed that MFAP5 over-expression accelerated the proliferative and metastatic properties of BLBC cells, and knockdown of MFAP5 had the opposite effect. Furthermore, we demonstrated that MFAP5 promoted the tumorigenesis and metastasis of BLBC in vivo by developing tumor xenograft and axillary lymph nodes, lung metastasis models. Specifically, qRT-PCR and Western blotting showed that MFAP5 upregulated the expression of N-cadherin and Type I Collagen and downregulated E-cadherin in BLBC cells. This provides evidence that MFAP5 promotes EMT in BLBC cells. Furthermore, similar results were found in xenograft mouse tumor sections, suggesting that MFAP5 promoted EMT in vivo.

EMT is a process that epithelial cells may transform into mesenchymal cells by losing cellular morphology and migratory capacity [36]. It plays an important role in the early stage of tumor metastasis and progression of cancer [37]. Identifying and understanding the signaling mechanisms that promote EMT may lead to novel therapeutic strategies to inhibit this cellular transformation in human cancer. There are several signal transduction pathways involved in the EMT process, such as transforming growth factor beta1 (TGF-β), NF-κB, Wnt, and Notch [38, 39]. It is reported that TGF-β signaling pathway participates in many biological events and in the process of regulation of these cellular biology events, TGF-β signal mainly interacts with other signaling pathways in order to obtain synergy or antagonism effect to exert its biological activities [40, 41]. Smad is a group of intracellular proteins that are essential for the transmission of TGF-β signals from the cell surface to the cell nucleus to promote the transcription of the target gene. A lot of evidences showed that TGF-β can stimulate EMT in cancer cells and promote distant metastasis by depending or not depending on Smad2/3 signaling pathways [42]. Notch has been shown to be involved in a variety of cellular processes including cell division, cell fate specification, differentiation, apoptosis, migration, invasion, adhesion, epithelial cell polarity, stem cell maintenance, and angiogenesis [43]. Several lines of evidence support a role of Notch activation in BLBC tumors [44]. In our results, we explored the mechanism of MFAP5 biological function in EMT. We detected MFAP5 can promote the phosphorylation of Smad2/Smad3. And the expression levels of Hes1 and Hes5, downstream effectors of Notch signaling pathway, were also increased. Inhibition of TGF-β and Notch signaling pathways, the expression of p-Smad2/p-Smad3, Hes1 and Hes5 were decreased. In addition, inhibition of the TGF-β and Notch signaling can also block the expression of EMT transcription factors including Snail, Slug, Zeb1, Zeb2 and Twist, which could induce epithelial differentiation. At the same time, MFAP5-mediated migration, invasion and proliferation of BT20 cells were blocked by TGF-β and Notch signaling pathways inhibitors. It implied that MFAP5 played an essential role in TGF-β and Notch signaling in BLBC pathogenesis. However, the downstream mechanism underlying the links between MFAP5 and EMT program remains relatively unknown.

Clearly, our study suggests that MFAP5 overexpression represents an oncogenic event that is responsible for the aggressive behaviors of BLBC cells. Thus, we assumed that MFAP5 might promote the procession of EMT by TGF-β/Notch signaling pathway. However, further studies are still needed to elucidate novel mechanisms or cellular cues for MFAP5 eliciting TGF-β/Notch signaling.

In summary, our findings reveal a novel gene MFAP5 involved in BLBC. It is a potential biomarker and a novel therapeutic target for BLBC patients at risk of metastasis. It plays an irreplaceable role in BLBC progression which may attribute to promoting EMT which enhances cell migration, invasion, and metastasis. Additional studies will be needed to elucidate the precise signaling pathway to provide important basis for using TGF-β and Notch signaling pathways. These findings may prove to be clinically useful for developing a new therapeutic target for BLBC invasion and metastasis.

## Additional files

**Additional file 1: Table S1.** The relative mRNA level of MFAP5 in BT20 and HS578T cells.

**Additional file 2: Figure S1.** The quantitative expression of MFAP5 mRNA in BT20 and HS578T cells after transfection. (A) BT20 cells transfected with MFAP5 lentivirus overexpressed MFAP5 compared to vehicle. **P < 0.01 vs LV-vehicle. (B) Compared to control shRNA, HS578T cells transfected with MFAP5 shRNA expressed decreased mRNA level of MFAP5. **P < 0.01 vs control shRNA.

**Additional file 3: Figure S2.** The inhibitory effect ofSB431542 and ly-411575 on TGF-β and Notch signaling pathways at different concentrations. BT20-LV-vehicle and BT20-LV-MFAP5 cells were treated with SB431542 or ly-411575 as indicated for 6 and 24 h, then the cells were collected to detect the expression of p-Smad2/Smad3, Hes1 and Hes5. (A) Immunofluorescence showed that SB431542 dose-dependently decreased the level of p-Smad2/Smad3 which was elevated by MFAP5. And at the concentration of 10 μM, SB431542 exhibited the largest inhibitory effect. (B) ly-411575 dose-dependently decreased the level of Hes1 and Hes5 induced by MFAP5 and the inhibitory effect was similar and more effective at 0.1 and 1 μM. **P < 0.01 vs corresponding LV-vehicle; ^##^*P* < 0.01 vs corresponding LV-MFAP5.
