# Supplementary material for: MFAP5 promotes basal-like breast cancer progression by activating the EMT program
Source: Cell Biosci. 2019 Mar 7;9:24. doi: 10.1186/s13578-019-0284-0 (PMC6407223; doi:10.1186/s13578-019-0284-0)
Supplement: Supplementary file 1 — Additional file 1: Table S1. The relative mRNA level of MFAP5 in BT20 and HS578T cells. [file 13578_2019_284_MOESM1_ESM.pdf]

	Target	Ct	C(t)Mean	C(t) Std.	Reference	Ct	C(t)Mean	C(t) Std.	$\triangle$Ct	$2^{-\triangle Ct}$
BT20	MFAP5	28.10			actin	13.36				
BT20	MFAP5	27.97	28.14	0.193	actin	13.60	13.50	0.123	14.64	3.908E-05
BT20	MFAP5	28.35			actin	13.53				
HS578T	MFAP5	24.58			actin	13.59				
HS578T	MFAP5	24.72	24.65	0.070	actin	13.75	13.67	0.080	10.98	4.940E-04
HS578T	MFAP5	24.65			actin	13.66				
