# Supplementary figures and images for: MFAP5 promotes basal-like breast cancer progression by activating the EMT program
Source: Cell Biosci. 2019 Mar 7;9:24. doi: 10.1186/s13578-019-0284-0 (PMC6407223; doi:10.1186/s13578-019-0284-0)

A

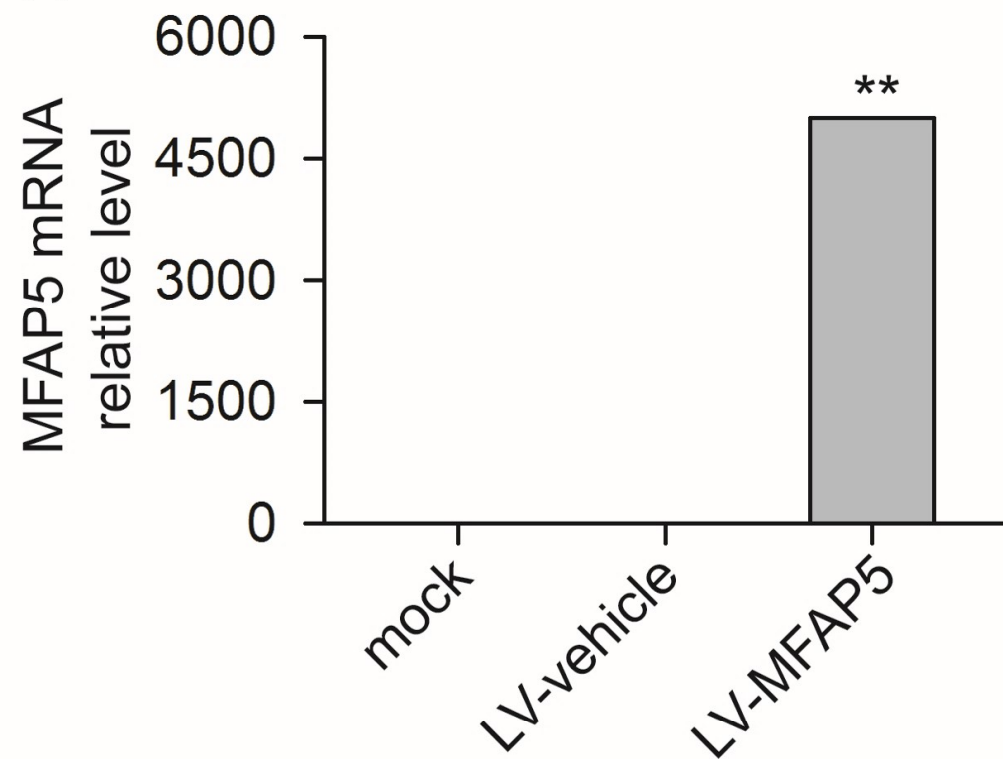

B

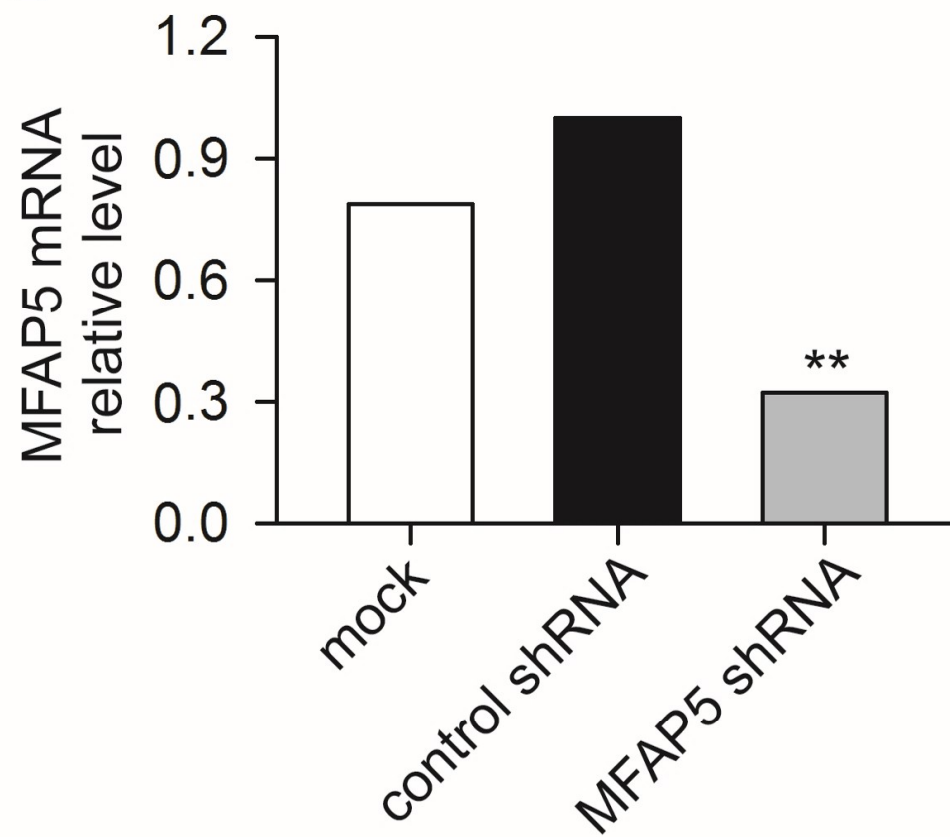

Supplement: Supplementary file 2 — Additional file 2: Figure S1. The quantitative expression of MFAP5 mRNA in BT20 and HS578T cells after transfection. (A) BT20 cells transfected with MFAP5 lentivirus overexpressed MFAP5 compared to vehicle. **P < 0.01 vs LV-vehicle. (B) Compared to control shRNA, HS578T cells transfected with MFAP5 shRNA expressed decreased mRNA level of MFAP5. **P < 0.01 vs control shRNA. [file 13578_2019_284_MOESM2_ESM.pdf]

A

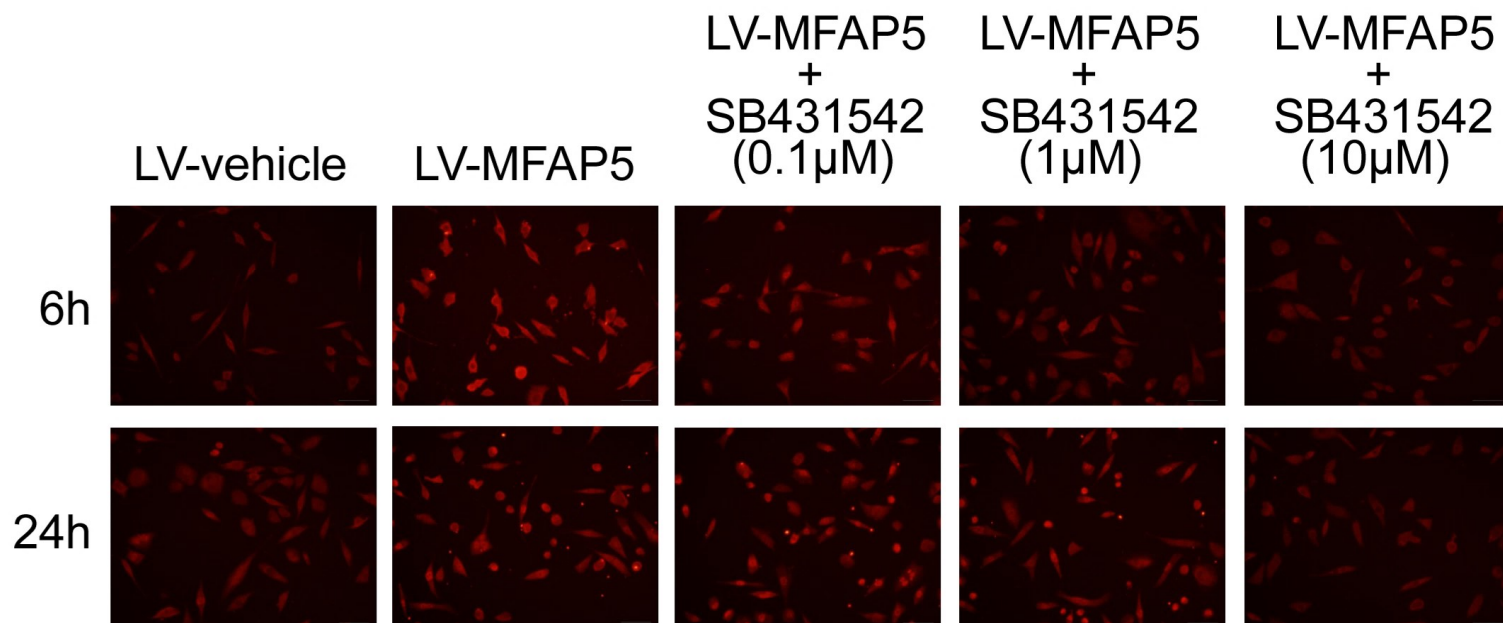

B

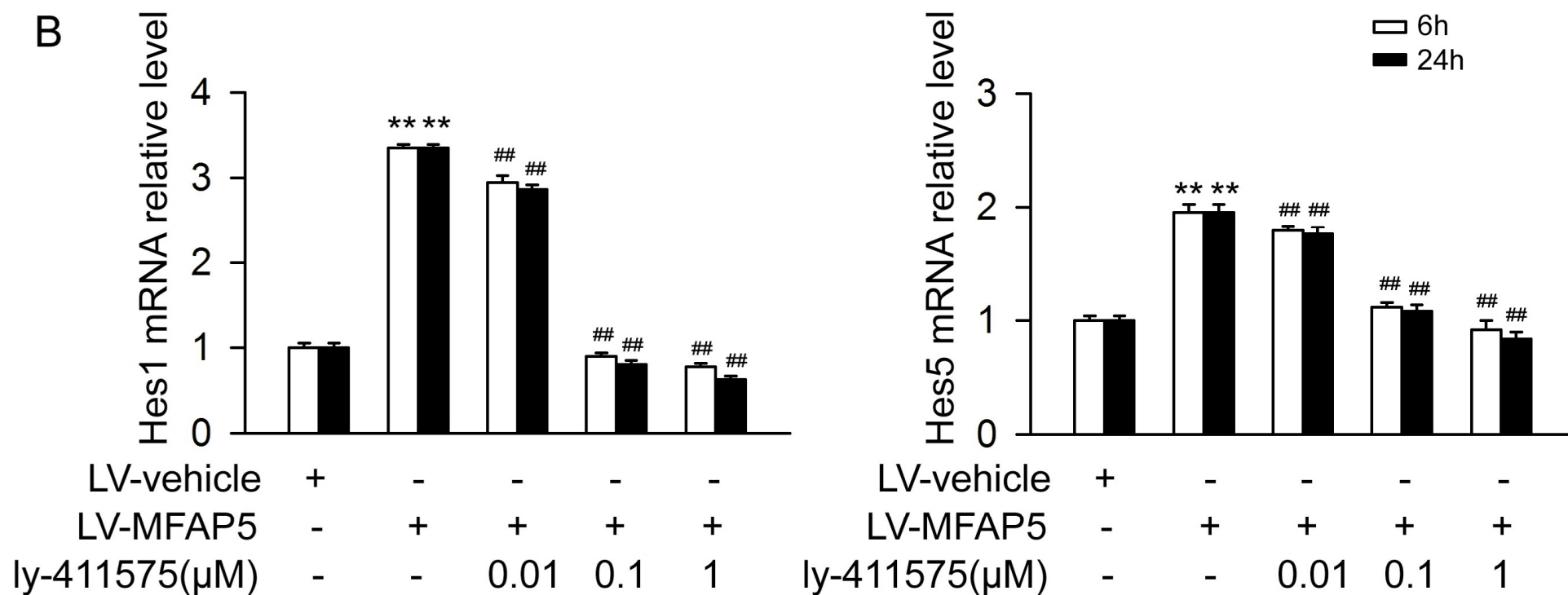

Supplement: Supplementary file 3 — Additional file 3: Figure S2. The inhibitory effect ofSB431542 and ly-411575 on TGF-β and Notch signaling pathways at different concentrations. BT20-LV-vehicle and BT20-LV-MFAP5 cells were treated with SB431542 or ly-411575 as indicated for 6 and 24 h, then the cells were collected to detect the expression of p-Smad2/Smad3, Hes1 and Hes5. (A) Immunofluorescence showed that SB431542 dose-dependently decreased the level of p-Smad2/Smad3 which was elevated by MFAP5. And at the concentration of 10 μM, SB431542 exhibited the largest inhibitory effect. (B) ly-411575 dose-dependently decreased the level of Hes1 and Hes5 induced by MFAP5 and the inhibitory effect was similar and more effective at 0.1 and 1 μM. **P < 0.01 vs corresponding LV-vehicle; ##P < 0.01 vs corresponding LV-MFAP5. [file 13578_2019_284_MOESM3_ESM.pdf]
